# Transplantation of Autologous Mesenchymal Stem Cells for End-Stage Liver Cirrhosis: A Meta-Analysis Based on Seven Controlled Trials

**DOI:** 10.1155/2015/908275

**Published:** 2015-03-15

**Authors:** Xiang-Rui Ma, Ya-Ling Tang, Ming Xuan, Zheng Chang, Xiao-Yi Wang, Xin-Hua Liang

**Affiliations:** ^1^State Key Laboratory of Oral Diseases, West China Hospital of Stomatology, Sichuan University, No. 14, 3rd Section, Renmin South Road, Chengdu, Sichuan 610041, China; ^2^Department of Oral and Maxillofacial Surgery, West China Hospital of Stomatology, Sichuan University, No. 14, 3rd Section, Renmin South Road, Chengdu, Sichuan 610041, China

## Abstract

*Background*. The bone marrow-derived mesenchymal stem cells (BM-MSCs) have demonstrated great potential as regenerative medicine in different therapeutic applications. This study aims to pool previous controlled clinical trials to make an update assessment of the effectiveness of BM-MSC transplantation on end-stage liver cirrhosis. *Methods*. Relevant studies published between January 1990 and June 2014 were searched among Pubmed, Embase, and ClinicalTrial.gov. A meta-analysis was performed to assess the effect of BM-MSCs on liver function indicators, including Models of End-Stage Liver Disease (MELD) score, serum albumin (g/L), total bilirubin (mg/dl), Prothrombin concentration (%), and alanine aminotransferase (ALT) (U/L). *Results*. BM-MSCs therapy could significantly improve liver function in patients with end-stage liver cirrhosis, in terms of MELD score, serum albumin, total bilirubin, and prothrombin concentration, at least during the half year after transplantation. *Conclusions*. Due to BM-MSCs' immunomodulatory functions and the potential to differentiate into hepatocytes, they are a promising therapeutic agent to liver cirrhosis. Considering currently available evidence, this therapy is relatively safe and effective in improving liver function. However, how different variables should be controlled to optimize the therapeutic effect is still not clear. Thus, future mechanism studies and clinical trials are required for this optimization.

## 1. Introduction

Cirrhosis is a common outcome of liver fibrosis caused by chronic liver diseases (CLD). This disease is characterized as reduced liver regeneration and liver dysfunction and can further lead to portal hypertension and end-stage liver disease (ESLD) [1]. It is the major cause of morbidity in patients with CLD. Alcohol abuse and infection of hepatitis B and C viruses cause the majority of cirrhosis across the world [2].

Currently, the most effective treatment for end-stage cirrhosis is liver transplantation. However, due to lack of organ donors, risk of rejection, various complications, and high cost, this treatment is quite limited in clinical practice [3]. In addition, if liver transplantation failed, there will be further extensive and progressive fibrosis, leading to further hamper of liver regeneration and irreversible cirrhosis [3, 4]. Thus, during the past decades, scholars have been making every effort to explore new techniques to stimulate liver regeneration.

The bone marrow is a reservoir of various stem cells. The bone marrow-derived mesenchymal stem cells (BM-MSCs) were found to have differentiative plasticity and demonstrated great potential as regenerative medicine in different therapeutic applications [5–8]. Actually, BM-MSCs presented the ability of mesodermal and neuroectodermal differentiation and thus can differentiate into functional hepatocyte-like cells [9]. In this respect, a series of studies have been performed to assess the application of BM-MSCs to promote liver regeneration and to alleviate cirrhosis. Some recent animal-based studies showed that BM-MSC transplantation could ameliorate liver fibrosis and improve liver functions [10, 11]. However, the effectiveness of this therapy in recent clinical trials is still conflicting. Several clinical trials demonstrated that the BM-MSC transplantation could significantly reverse hepatic failure with only limited side effects [12–14]. But some studies reported no significant benefits to liver function and survival [15]. Therefore, this study aims to pool previous controlled clinical trials to make an update assessment of the effectiveness of BM-MSC transplantation on end-stage liver cirrhosis.

## 2. Methods

### 2.1. Literature Search

Relevant studies published between January 1990 and June 2014 were searched among Pubmed, Embase, and ClinicalTrial.gov. The following terms and strategies are used to guide searching in these databases: (“bone marrow stem cell” OR “mesenchymal stem cell”) AND (“chronic liver disease” OR “cirrhosis”). No language restriction was set for searching. To avoid missing relevant and quailed trials, backward snowballing method was used for manually screening the reference lists of included studies, relevant meta-analysis, and reviews.

### 2.2. Inclusion and Exclusion Criteria

Clinical trials meeting the following criteria at the same time were included in this study. (1) Clinical trials involved end-stage liver cirrhosis patients; (2) studies assigned patients to autologous BM-MSCs therapy group and placebo or traditional supportive treatment group; (3) studies reported liver function outcomes in detailed data; studies with at least 1 month follow-up after cell transplantation. Studies meeting any of the following criteria were excluded: (1) case report, editorial, or letter to editors; (2) case series with only experimental arm; (3) studies that involved patients who had coexisting liver tumors, kidney or heart failure, infection of human immunodeficiency virus, and portal vein thrombosis and were pregnant.

### 2.3. Data Extraction, Study Quality, and Bias Assessment

The following basic information of study characteristics were extracted: last name of the first author, year of publication, country in which the study was conducted, cause of cirrhosis, number of patients in each group, type of MSCs used, the method of purity assessment, the number of cells transplanted, therapy frequency, the route of cell delivery, therapy in control group, and the maximum follow-up. To assess the effectiveness of BM-MSCs transplantation on liver function, original data of the following five indicators were extracted from the trials: Models of End Stage Liver Disease (MELD) score, serum albumin (g/L), total bilirubin (mg/dL), prothrombin concentration (%), and alanine aminotransferase (ALT) (U/L). Quality of the included trials was assessed by methodological quality item of controlled trials according to the Cochrane Handbook for Systematic Reviews of Interventions.

### 2.4. Data Synthesis and Analysis

RevMan 5.3 (Cochrane Collaboration) was used for data integration and analysis. All of the outcome indicators are discontinuous data. Thus the mean and SD data were extracted and pooled to make estimate of mean difference and corresponding 95% confidence intervals (CIs). To line up the comparisons, outcome measured after 1, 3, and 6 months of transplantation was extracted separately and used for stratified comparison. Between studies heterogeneity was assessed with Chi square-based *Q* test and *I* ^2^. *P* < 0.1 or *I* ^2^ > 50% donates significant heterogeneity. To identify suitable model of estimation, *P* value of *Q* test and *I* ^2^ was calculated in a primary analysis based on fixed-effects model. If *I* ^2^ ≤ 50% and *P* ≥ 0.1, fixed-effects model with Mantel–Haenszel method was used; otherwise random effects model was used. The significance of pooled estimates was assessed with *Z* test, in which *P* < 0.05 is considered as significant difference.

## 3. Results

### 3.1. Studies Included

**Figure 1:**
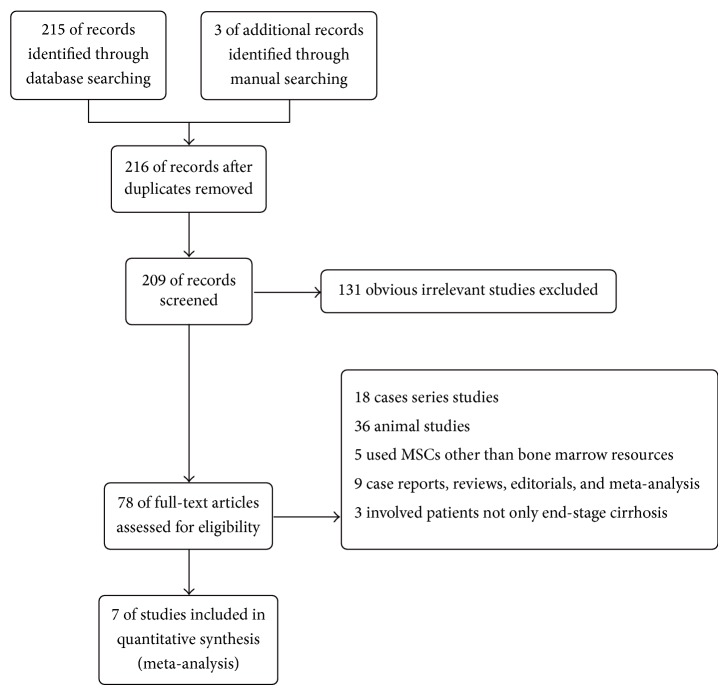
The searching and screening process.

Through searching in the databases, a total of seven trials [12–18] were included. The general searching and screening process is described in Figure 1. The basic information of the trials was summarized in Table 1. The seven studies were published between 2011 and 2014, with four performed in Egypt, two in China, and one in Iran. A total of 489 patients were included, 256 received BM-MSCs transplantation and 233 had placebo or traditional supportive treatment. The causes of cirrhosis mainly were hepatitis B or C infection. All of the studies used BM-MSCs. One study did not provide exact data of the number of cells infused [16]. In the remaining six studies, the number of cells infused varied from 10^6^/kg to 8.45 ± 3.28 × 10^8^. Three studies had cells transplanted intravenously [14, 15, 17], two through hepatic artery [16, 18], one through portal vein [13], and one through intrasplenic or intrahepatic route [12]. The follow-up period ranged from 6 months to 12 months. The quality assessment of the trials was concluded in Table 2. The quality of the trials was relatively low. Two studies were nonrandomized studies [14, 16]. Only one study has blind design [15].

### 3.2. The Effectiveness of BM-MSCs on MELD Score

**Figure 2:**
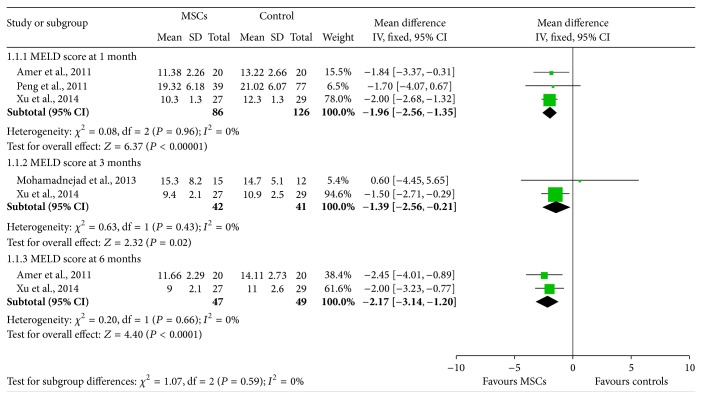
The effectiveness of BM-MSCs on MELD score.

Three [12, 16, 18], two [15, 18], and two [12, 18] studies assessed MELD score 1 month, 3 months, and 6 months after transplantations of BM-MSCs (Figure 2). Generally, BM-MSCs therapy was associated with significantly lower MELD score at 1 month (WMD: −1.95, 95% CI: −2.56 to −1.35, *P* < 0.00001), 3 months (WMD: −1.39, 95% CI: −2.56 to −0.21, *P* = 0.02), and 6 months (WMD: −2.17, 95% CI: −3.14 to −1.20, *P* < 0.0001) (Figure 2). No significant heterogeneity was observed in any of the three groups, suggesting a consistent effect of BM-MSCs during the follow-up period.

### 3.3. The Effectiveness of BM-MSCs on Serum Albumin

**Figure 3:**
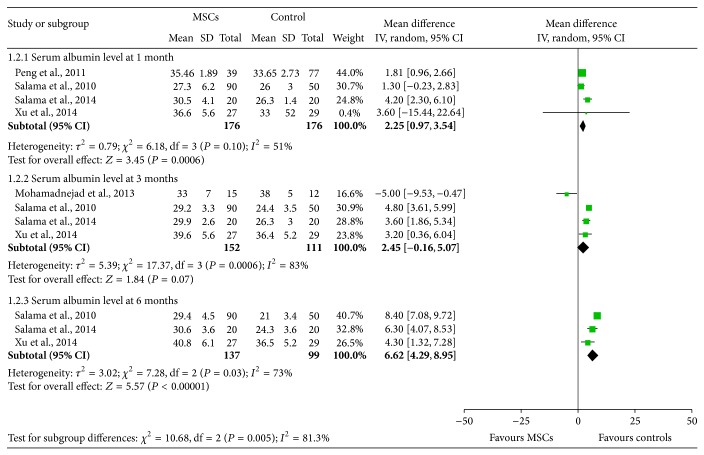
The effectiveness of BM-MSCs on serum albumin.

Four [13, 16–18], four [13, 15, 17, 18], and three [12, 16, 17] studies assessed serum albumin 1 month, 3 months, and 6 months after transplantations of BM-MSCs (Figure 3). Generally, BM-MSCs therapy was associated with significantly higher serum albumin at 1 month (WMD: 2.25, 95% CI: 0.97 to 3.54, *P* = 0.0006), 3 months (WMD: 2.45, 95% CI: −0.16 to 5.07, *P* = 0.07), and 6 months (WMD: 6.62, 95% CI: 4.29 to 8.95, *P* < 0.00001). However, significant heterogeneity was observed in the three groups (*I* ^2^ = 51%, 83%, and 73%, resp.) (Figure 3). In months 1 and 6 measurement, all of the studies reported similar serum albumin increasing trend in BM-MSCs groups. The heterogeneity was mainly related to different level of positive outcome. However, in month 3 measurement, Mohamadnejad et al. [15] reported contracting results, which observed that BM-MSCs therapy was associated with decreased serum albumin. Exclusion of this study could decrease the heterogeneity to a nonsignificant level.

### 3.4. The Effectiveness of BM-MSCs on Total Bilirubin

**Figure 4:**
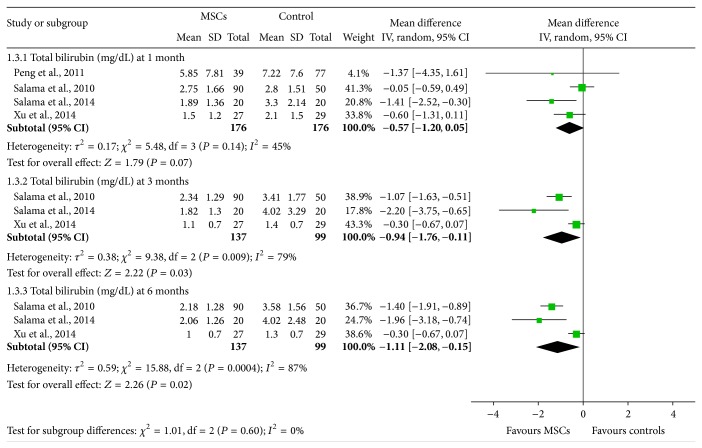
The effectiveness of BM-MSCs on total bilirubin.

Four [13, 16–18], three [15–17], and three [15–17] studies assessed total serum bilirubin 1 month, 3 months, and 6 months after transplantations of BM-MSCs (Figure 4). Generally, BM-MSCs therapy was associated with moderate serum bilirubin reduction at 1 month (WMD: −0.57, 95% CI: −1.20 to 0.05, *P* = 0.07), 3 months (WMD: −0.94, 95% CI: −1.76 to −0.11, *P* = 0.03), and 6 months (WMD: −1.11, 95% CI: −2.08 to −0.15, *P* = 0.0004). However, significant heterogeneity was observed at 3 and 6 months measurement (*I* ^2^ = 79% and 87%, resp.) (Figure 4). However, all of the studies in these two measurements reported similar serum bilirubin decreasing trend in BM-MSCs groups. The heterogeneity was mainly related to different level of positive outcome.

### 3.5. The Effectiveness of BM-MSCs on Prothrombin Concentration

**Figure 5:**
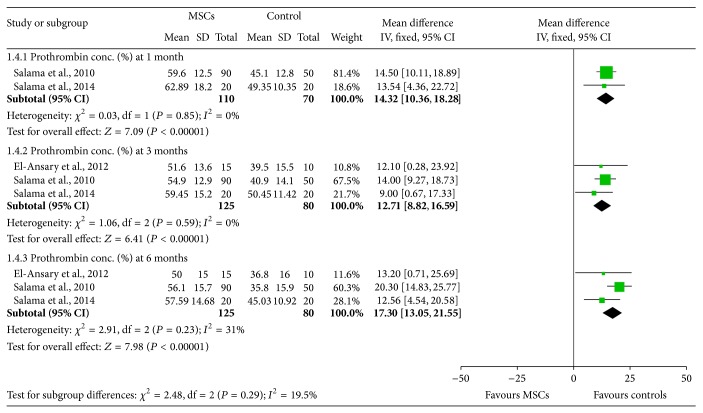
The effectiveness of BM-MSCs on prothrombin concentration.

Two [13, 17], three [13, 14, 17], and three [13, 14, 17] studies assessed prothrombin concentration 1 month, 3 months, and 6 months after transplantations of BM-MSCs (Figure 5). Generally, BM-MSCs therapy was associated with significantly increased prothrombin concentration at 1 month (WMD: 14.32, 95% CI: 10.36 to 18.28, *P* < 0.00001), 3 months (WMD: 12.71, 95% CI: 8.82 to 16.59, *P* < 0.00001), and 6 months (WMD: 17.30, 95% CI: 13.05 to 21.55, *P* < 0.00001) (Figure 5). Findings are highly consistent in these studies. No significant heterogeneity was observed in the three groups.

### 3.6. The Effectiveness of BM-MSCs on Alanine Aminotransferase

**Figure 6:**
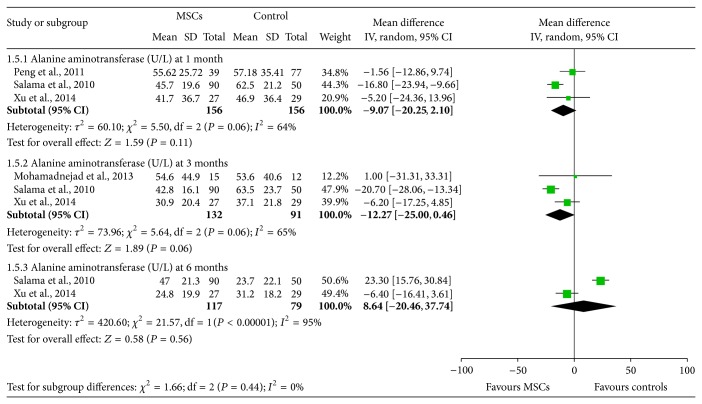
The effectiveness of BM-MSCs on alanine aminotransferase.

Three [13, 16, 18], three [13, 15, 18], and two [14, 17] studies assessed alanine aminotransferase 1 month, 3 months, and 6 months after transplantations of BM-MSCs (Figure 6). Generally, the effect of BM-MSCs therapy on lowering ALT was significant at 1 month (WMD: −9.07, 95% CI: −20.25 to 2.10, *P* = 0.11) and 3 months (WMD: −12.27, 95% CI: −25.00 to 0.46, *P* = 0.06), but not at 6 months (WMD: 8.64, 95% CI: −20.46 to 37.74, *P* = 0.56) (Figure 6). Findings are highly inconsistent in these studies. Significant heterogeneity was observed in the three groups (*I* ^2^ = 64%, 65%, and 95%, resp.).

## 4. Discussion

Cirrhosis is a common final pathologic outcome of chronic liver diseases. The ideal strategy to treat liver cirrhosis is to regenerate new hepatocytes as replacement to the damaged cells, without excessive fibrosis. Up till now, liver transplantation has been considered as the only effective curative treatment for decompensated cirrhosis [19]. However, these procedures have limited use due to lack of donors, high cost, and technical difficulties [19]. Recent MSCs-based cell therapy has demonstrated great potential for tissue repair in animal studies, giving rise to the hope of successful regenerative hepatology. Although one recent meta-analysis assessed transplantation of MSCs for liver cirrhosis [20], it only recruited two controlled trials (only 61 patients in total) and three single arm studies, which means their comparison was mainly based on two small studies and with limited statistical power. It is not appropriate to make conclusions based on such a small sample base. In fact, there are four new controlled trials published in 2013 and 2014 providing new evidence. Thus, an updated meta-analysis is necessary. This meta-analysis based on seven controlled clinical trials which included 489 patients demonstrated that BM-MSCs therapy could significantly improve liver function, in terms of MELD score, serum albumin, total bilirubin, and prothrombin concentration.

Based on previous studies, BM-MSCs could regulate fibrogenetic process through the following processes: inhibiting proliferation of hepatic stellate cells (HSCs), promoting HSC apoptosis; stimulating endogenous hepatocyte regeneration; inhibiting ECM accumulation and hepatocyte-like differentiation [21, 22]. These therapeutic effects are mainly mediated by their release of trophic and immunomodulatory factors, changing the behavior of hepatic stellate cells that are critical in the development of liver fibrosis. For example, MSCs can secrete IL-10 after transplantation, which contributes to reduced proliferation of stellate cells and collagen type I synthesis [23]. Through secreting HGF and nerve growth factor (NGF), BM-MSCs induce apoptosis of HSCs [23, 24]. In addition, MSCs can also alleviate cirrhosis through expressing matrix metalloproteinase-9 (MMP-9), which has antifibrotic effect through degrading the extracellular matrix [25]. Besides the mechanism of paracrine, there are also some minor mechanisms involved. Due to the genomic plasticity and inducing effect of microenvironment, a small proportion of BM-MSCs could cause transdifferentiation of stem cells into functional hepatocytes [26, 27]. In addition, some scholar indicated that BM-MSCs also could infuse with host cells, as a source of bone marrow-derived hepatocytes [28, 29].

However, the effectiveness of MSCs therapy is affected by a wide range of factors, including the number of cells transplanted, the cytokines and growth factor added in culture media, and the administration route as well as the supportive care after treatment. For example, Salama et al. [17] gave patients 300 *μ*g granulocyte colony-stimulating factor (G-CSF) daily for 5 days before transplantation of BM-MSCs. This agent is helpful at mobilizing BM-MSCs into the peripheral blood and promoting homing into the liver [30, 31]. Amer's study had the BM-MSCs treated with HGF, as an induction of hepatocyte-like cells [12]. Salama et al. selectively used CD34+ and CD133+ BM-MSCs, which have strong stem cell characteristics [13]. However, how these variables influence the therapeutic effects is still not quite clear. Due to the limited number of studies included and inconsistent use of outcome indicators, it is not possible in this study to make stratified analysis to explore the influence of these variables. Actually, these variables are quite important factors when optimizing the therapy. For example, intravenously injected BM-MSCs only migrate into normal or injured liver parenchyma under chronic injury. In acutely injured livers, the transplanted cells might differentiate into myofibroblasts, rather than into hepatocytes [32]. In addition, the route of the transplantation may also influence the myofibroblastic differentiation and engraftment of the transplanted MSCs. intrahepatic injection might increase the ratio of myofibroblasts differentiation, while intrasplenic injection could not achieve stable engraftment [33]. To avoid the unwanted differentiation, several studies suggest that BM-MSCs should be better differentiated into hepatocyte-like cells in vitro before transplantation [34]. Considering the influence of these variables on therapeutic effect, large randomized controlled trials with long-term follow-up are required for improvement and optimization of this therapy.

This study also has several limitations. Firstly, the number of trials included and the number of participants in each of the trials were relatively small. Secondly, the quality of the trials is relatively low. Thirdly, the outcome indicators and the time of measurements were not consistent in the trials. Therefore, when pooling the findings, only limited number of studies were pooled when assessing certain outcome. Due to these limitations, the statistical power might not be strong enough to make confirmative conclusions. Fourthly, this study only included studies concerning BM-MSCs. In fact, transplantation of other of MSCs, such as adipose tissue-derived MSCs, has also been considered as potential treatment for liver failure [35, 36]. Compared with BM-MSCs, adipose tissue-derived MSCs are more abundant, proliferate better, and are more similar to hepatocytes [37]. Therefore, it is quite necessary to further assess the application of different MSCs in the future.

## 5. Conclusions

Due to BM-MSCs' immunomodulatory functions and the potential to differentiate into hepatocytes, they are promising therapeutic agents to liver cirrhosis. Considering current available evidence, this therapy is relatively safe and effective in improving liver function. However, future mechanism studies and clinical trials are required for optimizing the therapeutic effects.

## Figures and Tables

**Table 1:** The key characteristics of trials included.

Study	Country	Cause of cirrhosis	Number of patients		Purity assessment	Type of MSC	Number of cells transplanted^*^	Therapy frequency	Route	Control therapy	Maximum follow-up
			I	C							
Salama et al., 2010 [13]	Egypt	Mixed	90	50	IMP	CD34+ and CD133+ aBM-MSCs	0.5 × 10^8^	Once	Portal vein	TST	6 months
Amer et al., 2011 [12]	Egypt	Hepatitis C	20	20	IP	aBM-MSCs stimulated with HGF	2 × 10^8^	Once	Intrasplenic or intrahepatic	TST	6 months
Peng et al., 2011 [16]	China	Hepatitis B	39	77	FC	aBM-MSCs	N.A.	Once	Hepatic artery	TST	12 months
El-Ansary et al., 2012 [14]	Egypt	Hepatitis C	15	10	FC	aBM-MSCs	10^6^/Kg	Once	Intravenous	TST	6 months
Mohamadnejad et al., 2013 [15]	Iran	Mixed	15	12	FC	aBM-MSCs	1.2–2.95 × 10^8^	Once	Intravenous	Placebo	12 months
Xu et al., 2014 [18]	China	Hepatitis B	27	29	FC	aBM-MSCs	8.45 ± 3.28 × 10^8^	Once	Hepatic artery	TST	6 months
Salama et al., 2014 [17]	Egypt	Hepatitis C	20	20	FC	aBM-MSCs	0.5 × 10^8^	Once	Intravenous	TST	6 months

I = intervention; C = control; MSC = mesenchymal stem cell; aBM-MSCs = autologous BM-MSCs; TST: traditional supportive treatment; IP = immunophenotyping; FC = flow cytometry; IMP = immunomagnetic purification; ^*^estimation according to delivery method.

**Table 2:** Quality assessments of trials included.

Study/quality components	Adequate random sequence generation (selection bias)	Adequate method of allocation concealment (selection bias)	Blinding of participants and personnel (performance bias)	Blinding of outcome assessment (detection bias)	Incomplete outcome data (attrition bias)	Selective reporting (reporting bias)
Salama et al., 2010 [13]	?	?	?	?	?	Y
Amer et al., 2011 [12]	Y	Y	?	?	Y	Y
Peng et al., 2011 [16]	N	N	?	?	?	Y
El-Ansary et al., 2012 [14]	N	N	?	?	?	Y
Mohamadnejad et al., 2013 [15]	Y	Y	Y	N	?	Y
Xu et al., 2014 [18]	Y	Y	?	?	?	Y
Salama et al., 2014 [17]	?	?	?	?	?	Y

“Y” indicating low risk of bias; “N” indicating high risk of bias; “?” indicating insufficient data for judgment.

## References

[1] Asrani S. K., Kamath P. S. (2013). Natural history of cirrhosis. *Current Gastroenterology Reports*.

[2] D'Amico G., Garcia-Tsao G., Pagliaro L. (2006). Natural history and prognostic indicators of survival in cirrhosis: a systematic review of 118 studies. *Journal of Hepatology*.

[3] Lucey M. R., Terrault N., Ojo L. (2013). Long-term management of the successful adult liver transplant: 2012 practice guideline by the American Association for the Study of Liver Diseases and the American Society of Transplantation. *Liver Transplantation*.

[4] Pinzani M., Rosselli M., Zuckermann M. (2011). Liver cirrhosis. *Best Practice & Research: Clinical Gastroenterology*.

[5] Volarevic V., Arsenijevic N., Lukic M. L., Stojkovic M. (2011). Concise review: mesenchymal stem cell treatment of the complications of diabetes mellitus. *Stem Cells*.

[6] Fule Robles J. D., Cheuk D. K., Ha S. Y., Chiang A. K., Chan G. C. (2014). Human herpesvirus types 6 and 7 infection in pediatric hematopoietic stem cell transplant recipients. *Annals of Transplantation*.

[7] Xiao C., Zhou S., Liu Y., Hu H. (2014). Efficacy and safety of bone marrow cell transplantation for chronic ischemic heart disease: a meta-analysis. *Medical Science Monitor*.

[8] Schwarz S., Huss R., Schulz-Siegmund M. (2014). Bone marrow-derived mesenchymal stem cells migrate to healthy and damaged salivary glands following stem cell infusion. *International Journal of Oral Science*.

[9] Zhang Z., Wang F.-S. (2013). Stem cell therapies for liver failure and cirrhosis. *Journal of Hepatology*.

[10] Wei T., Lv Y. (2013). Immediate intraportal transplantation of human bone marrow mesenchymal stem cells prevents death from fulminant hepatic failure in pigs. *Hepatology*.

[11] Shao C.-H., Chen S.-L., Dong T.-F. (2014). Transplantation of bone marrow-derived mesenchymal stem cells after regional hepatic irradiation ameliorates thioacetamide-induced liver fibrosis in rats. *The Journal of Surgical Research*.

[12] Amer M.-E. M., El-Sayed S. Z., El-Kheir W. A. (2011). Clinical and laboratory evaluation of patients with end-stage liver cell failure injected with bone marrow-derived hepatocyte-like cells. *European Journal of Gastroenterology & Hepatology*.

[13] Salama H., Zekri A.-R. N., Bahnassy A. A. (2010). Autologous CD34+ and CD133+ stem cells transplantation in patients with end stage liver disease. *World Journal of Gastroenterology*.

[14] El-Ansary M., Abdel-Aziz I., Mogawer S. (2012). Phase II trial: undifferentiated versus differentiated autologous mesenchymal stem cells transplantation in Egyptian patients with HCV induced liver cirrhosis. *Stem Cell Reviews and Reports*.

[15] Mohamadnejad M., Alimoghaddam K., Bagheri M. (2013). Randomized placebo-controlled trial of mesenchymal stem cell transplantation in decompensated cirrhosis. *Liver International*.

[16] Peng L., Xie D.-Y., Lin B.-L. (2011). Autologous bone marrow mesenchymal stem cell transplantation in liver failure patients caused by hepatitis B: short-term and long-term outcomes. *Hepatology*.

[17] Salama H., Zekri A. R., Medhat E. (2014). Peripheral vein infusion of autologous mesenchymal stem cells in Egyptian HCV-positive patients with end-stage liver disease. *Stem Cell Research & Therapy*.

[18] Xu L., Gong Y., Wang B. (2014). Randomized trial of autologous bone marrow mesenchymal stem cells transplantation for hepatitis B virus cirrhosis: regulation of Treg/Th17 cells. *Journal of Gastroenterology and Hepatology*.

[19] Murray K. F., Carithers R. L. (2005). AASLD practice guidelines: evaluation of the patient for liver transplantation. *Hepatology*.

[20] Pan X.-N., Zheng L.-Q., Lai X.-H. (2014). Bone marrow-derived mesenchymal stem cell therapy for decompensated liver cirrhosis: a meta-analysis. *World Journal of Gastroenterology*.

[21] Van Poll D., Parekkadan B., Cho C. H. (2008). Mesenchymal stem cell-derived molecules directly modulate hepatocellular death and regeneration in vitro and in vivo. *Hepatology*.

[22] Zhang B., Inagaki M., Jiang B. (2009). Effects of bone marrow and hepatocyte transplantation on liver injury. *Journal of Surgical Research*.

[23] Parekkadan B., van Poll D., Megeed Z. (2007). Immunomodulation of activated hepatic stellate cells by mesenchymal stem cells. *Biochemical and Biophysical Research Communications*.

[24] Lin N., Hu K., Chen S. (2009). Nerve growth factor-mediated paracrine regulation of hepatic stellate cells by multipotent mesenchymal stromal cells. *Life Sciences*.

[25] Higashiyama R., Inagaki Y., Hong Y. Y. (2007). Bone marrow-derived cells express matrix metalloproteinases and contribute to regression of liver fibrosis in mice. *Hepatology*.

[26] Jang Y.-Y., Collector M. I., Baylin S. B., Diehl A. M., Sharkis S. J. (2004). Hematopoietic stem cells convert into liver cells within days without fusion. *Nature Cell Biology*.

[27] Li T., Zhu J., Ma K. (2013). Autologous bone marrow-derived mesenchymal stem cell transplantation promotes liver regeneration after portal vein embolization in cirrhotic rats. *The Journal of Surgical Research*.

[28] Wang X., Willenbring H., Akkari Y. (2003). Cell fusion is the principal source of bone-marrow-derived hepatocytes. *Nature*.

[29] Vassilopoulos G., Wang P.-R., Russell D. W. (2003). Transplanted bone marrow regenerates liver by cell fusion. *Nature*.

[30] Christensen E. (2004). Prognostic models including the Child-Pugh, MELD and Mayo risk scores—where are we and where should we go?. *Journal of Hepatology*.

[31] Wylezoł I., Snarski E., Markiewicz M., Kyrcz-Krzemień S., Jedrzejczak W.-W., Walewski J. (2013). Comparision of benefits of early, delayed, and no administration of G-CSF after autologous peripheral blood stem cell transplantation in lymphoma patients. *Annals of Transplantation*.

[32] di Bonzo L. V., Ferrero I., Cravanzola C. (2008). Human mesenchymal stem cells as a two-edged sword in hepatic regenerative medicine: engraftment and hepatocyte differentiation versus profibrogenic potential. *Gut*.

[33] Baertschiger R. M., Serre-Beinier V., Morel P. (2009). Fibrogenic potential of human multipotent mesenchymal stromal cells in injured liver. *PLoS ONE*.

[34] Aurich H., Sgodda M., Kaltwaßer P. (2009). Hepatocyte differentiation of mesenchymal stem cells from human adipose tissue in vitro promotes hepatic integration in vivo. *Gut*.

[35] Fitzpatrick E., Wu Y., Dhadda P. (2015). Coculture with mesenchymal stem cells results in improved viability and function of human hepatocytes. *Cell Transplantation*.

[36] Salomone F., Barbagallo I., Puzzo L., Piazza C., Li Volti G. (2013). Efficacy of adipose tissue-mesenchymal stem cell transplantation in rats with acetaminophen liver injury. *Stem Cell Research*.

[37] Ochiya T., Yamamoto Y., Banas A. (2010). Commitment of stem cells into functional hepatocytes. *Differentiation*.

